# Supplementary material for: Genome-Wide Analysis of the FBA Subfamily of the Poplar F-Box Gene Family and Its Role under Drought Stress
Source: Int J Mol Sci. 2023 Mar 2;24(5):4823. doi: 10.3390/ijms24054823 (PMC10002531; doi:10.3390/ijms24054823)
Supplement: Supplementary file 1 [file ijms-24-04823-s001.zip › Fig.S1-s6.pdf]

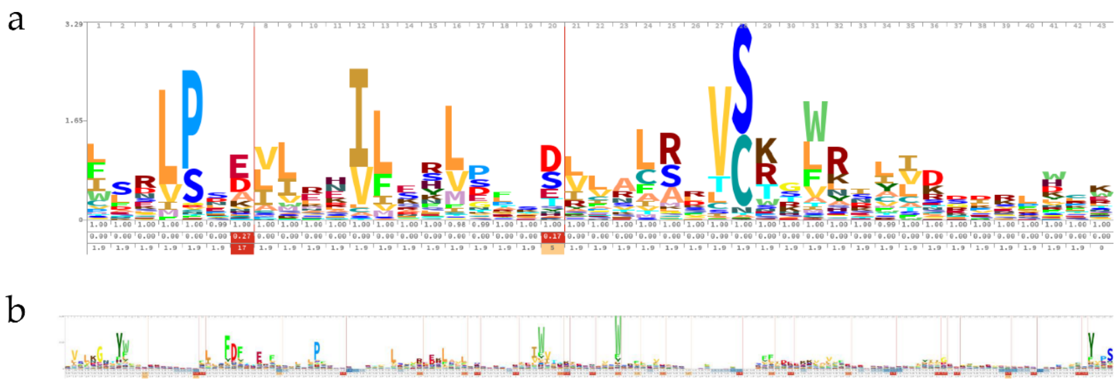

Figure S1. F-box and FBA1 motif sequencing of all proteins in *P. trichocarpa*. (a) F-box motif sequencing. (b) FBA1 motif sequencing. Numbers on the x-axis represent sequence positions in the F-box and FBA1 pattern. The numbers on the y-axis represent information content measured in bits. Serial logos are generated using the WebLogo tool.

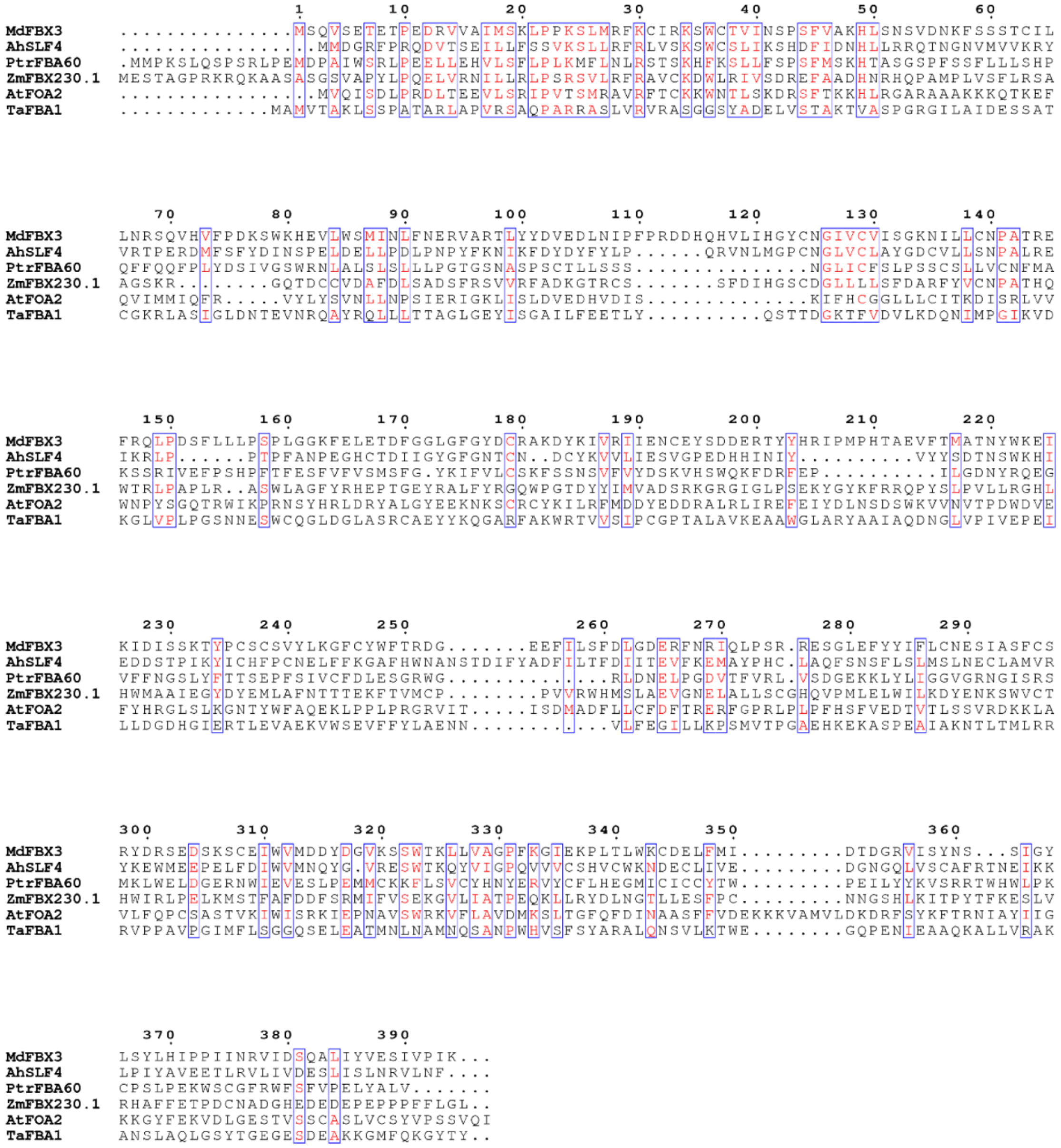

Figure S2. Representative FBA protein sequence alignment of each species. Using CLUSTALX to compare *Malus domestica* FBA protein *MdFBX3*, *Antirrhineum hispanicum* FBA protein *AhSLF4*, *Populus trichocarpa* FBA protein *PtrFBA60*, *Zea mays* FBA protein *FBX230.1*, *Arabidopsis thaliana* FBA protein *AtFOA2*, *Triticum aestivum* FBA protein *TaFBA1*.

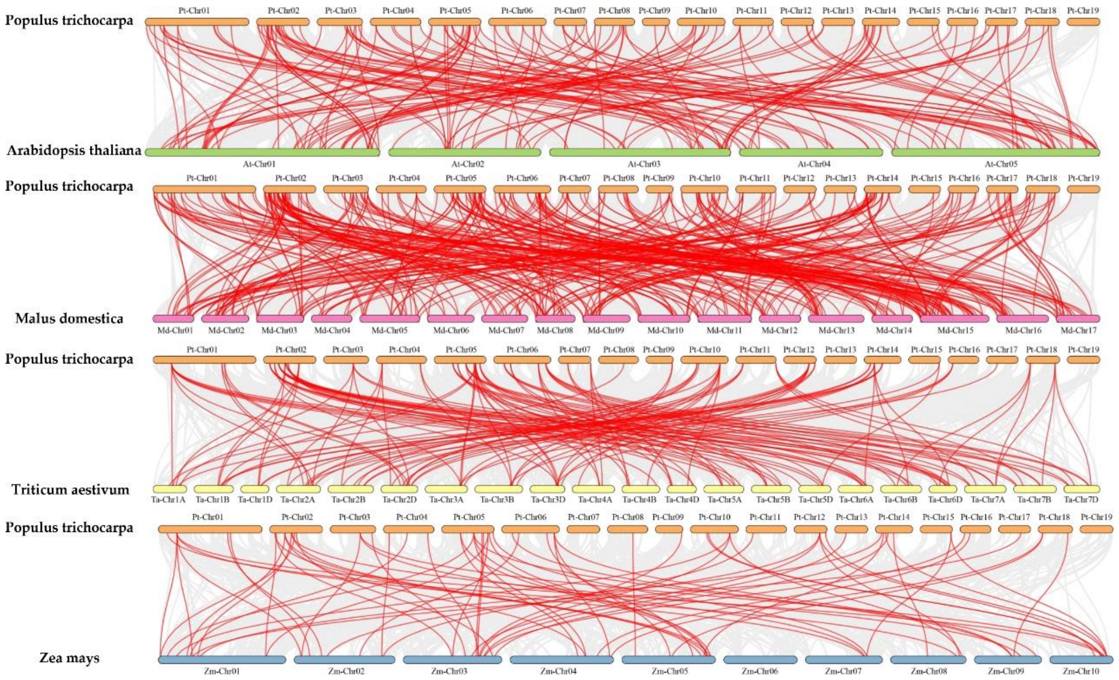

Figure S3. Collinear analysis of poplar F-box gene family with 4 typical plants (Arabidopsis, wheat, maize, apple). Gray lines in the background represent collinear blocks of *P. trichocarpa* and other species genomes, while red lines emphasize collinear PtrFBXs gene pairs.

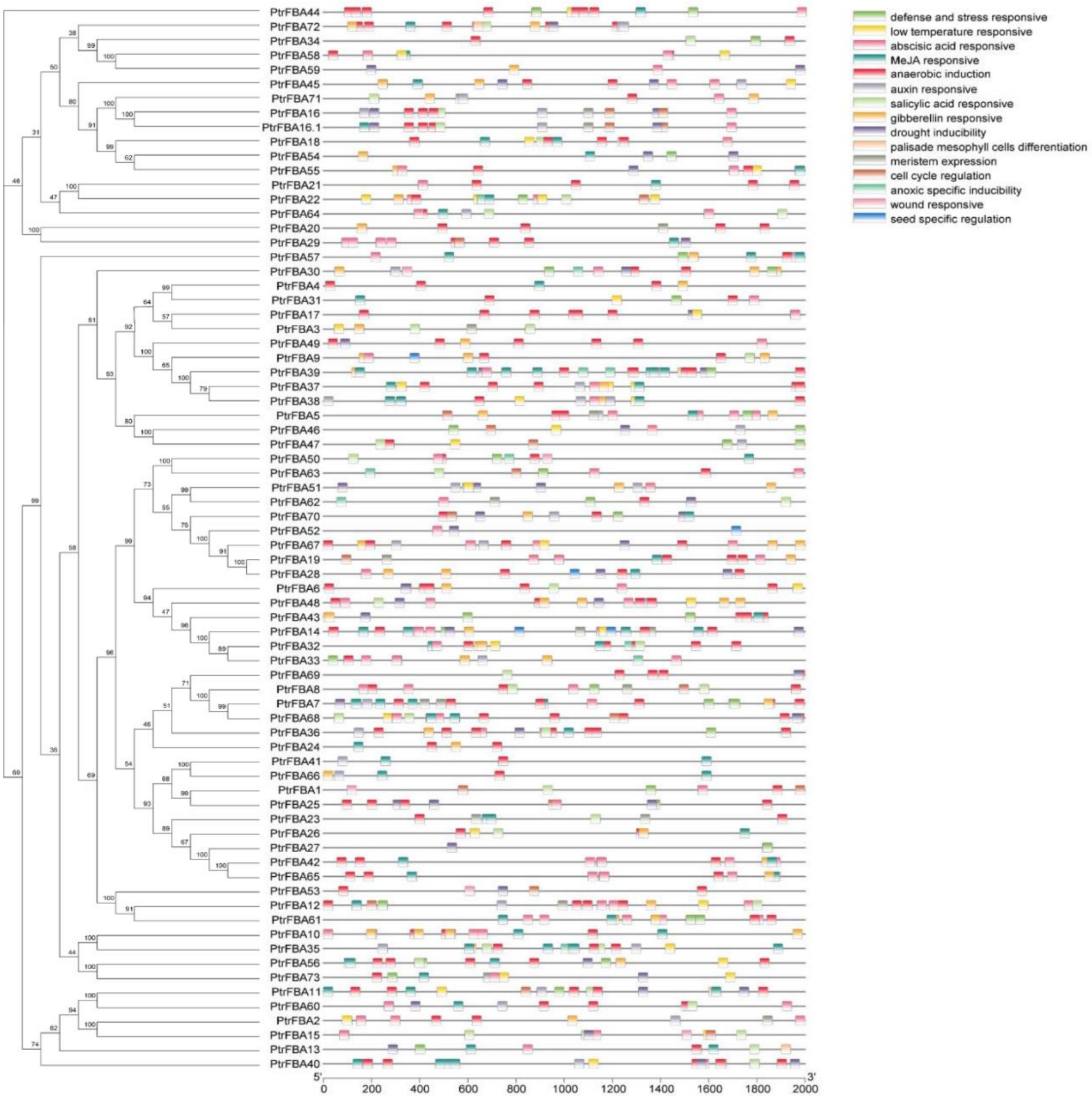

Figure S4. Cis-elemental analysis in the PtrFBAs gene family of *P. trichocarpa*. Relative positions of stress- and growth-associated cis-elements in the promoter regions of PtrFBAs. Different colors represent different cis-acting elements, and their positions correspond to the corresponding positions of the promoters.

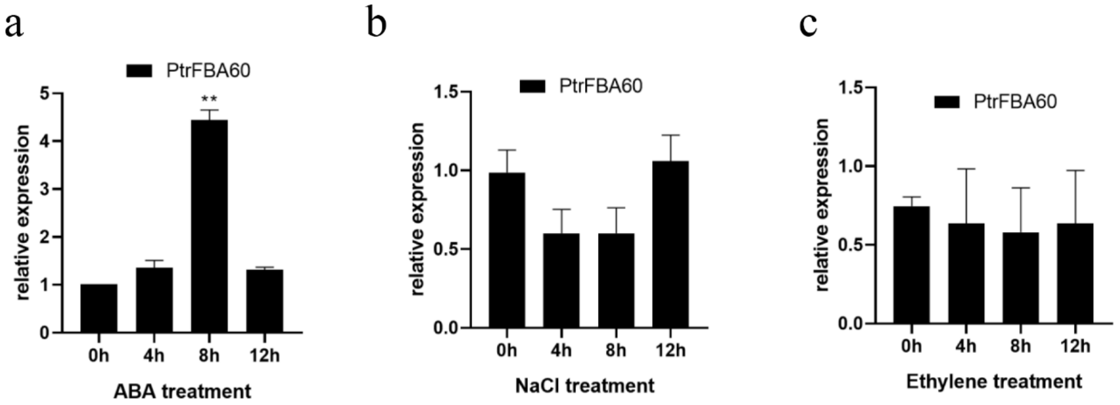

Figure S5. Expression patterns of PtrFBA60 under different treatments. (a) *PtrFBA60* expression pattern under ABA treatment. (b) *PtrFBA60* expression pattern under 200mM/LNaCl treatment. (c) *PtrFBA60* expression pattern under Ethylene treatment. Multiple comparisons were evaluated based on the least significant difference (LSD) test to calculate p-values (Asterisks denoted significant differences: * $p \leq 0.05$; *** $p \leq 0.01$).

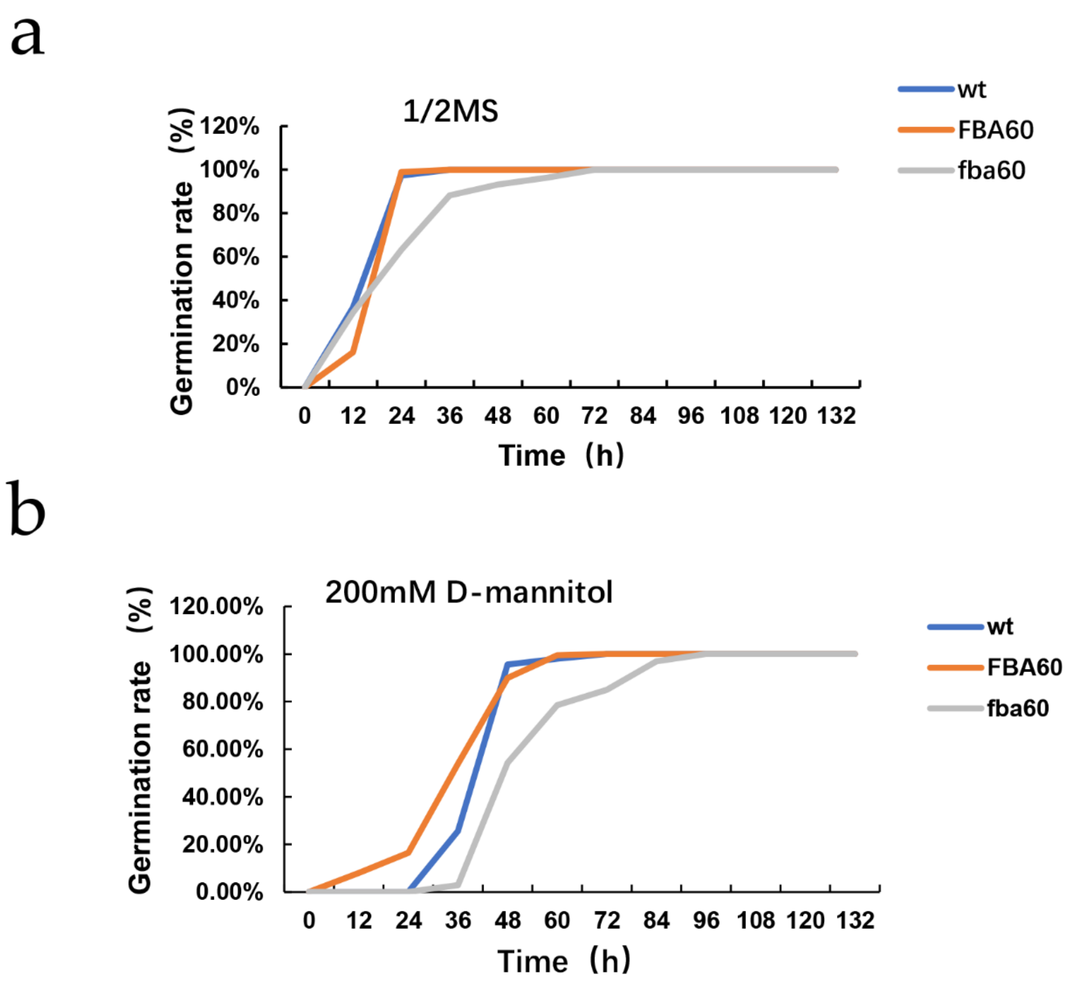

Fig S6. Germination rates of transgenic and wild-type Arabidopsis under mannitol treatment (a) Germination rate of different strains of Arabidopsis on 1/2MS medium(b) Germination rate of different strains of Arabidopsis thaliana supplemented with 200mM/L D-mannitol1/2MS medium.
